# Three-dimensional methodology for photogrammetric acquisition of the soft tissues of the face: a new clinical-instrumental protocol

**DOI:** 10.1186/2196-1042-14-32

**Published:** 2013-09-20

**Authors:** Roberto Deli, Luigi M Galantucci, Alberto Laino, Raoul D’Alessio, Eliana Di Gioia, Carmela Savastano, Fulvio Lavecchia, Gianluca Percoco

**Affiliations:** Università Cattolica del Sacro Cuore di Roma, Rome, 00198 Italy; Laboratorio di Prototipazione Rapida e Reverse Engineering, Dipartimento di Meccanica Matematica e Management, Politecnico di Bari, Bari, 70126 Italy; Università degli Studi di Napoli Federico II, Naples, 80138 Italy; Studio Associato di Odontoiatria dei Dottori Di Gioia, Bari, 70122 Italy; Studio di Odontoiatria Dottoressa Carmela Savastano, Florence, 50121 Italy; Polishape 3D srl, Bari, 70126 Italy

**Keywords:** Photogrammetric face scanner, Anthropometry, Facial soft tissue, 3D measurements, Aesthetic analysis

## Abstract

**Background:**

The objective of this study is to define an acquisition protocol that is clear, precise, repeatable, simple, fast and that is useful for analysis of the anthropometric characteristics of the soft tissue of the face.

**Methods:**

The analysis was carried out according to a new clinical-instrumental protocol that comprises four distinct phases: (1) setup of portable equipment in the space in which field analysis will be performed, (2) preparation of the subject and spatial positioning, (3) scanning of the subject with different facial expressions, and (4) treatment and processing of data. The protocol was tested on a sample comprising 66 female subjects (64 Caucasian, 1 Ethiopian, and 1 Brazilian) who were the finalists of an Italian national beauty contest in 2010. To illustrate the potential of the method, we report here the measurements and full analysis that were carried out on the facial model of one of the subjects who was scanned.

**Results:**

This new protocol for the acquisition of faces is shown to be fast (phase 1, about 1 h; phase 2, about 1.5 min; phase 3, about 1.5 min; phase 4, about 15 min), simple (phases 1 to 3 requiring a short operator training period; only phase 4 requires expert operators), repeatable (with direct palpation of anatomical landmarks and marking of their positions on the face, the problem of identification of these same landmarks on the digital model is solved), reliable and precise (average precision of measurements, 0.5 to 0.6 mm over the entire surface of the face).

**Conclusions:**

This standardization allows the mapping of the subjects to be carried out following the same conditions in a reliable and fast process for all of the subjects scanned.

## Background

The need for three-dimensional (3D), noninvasive methods and diagnostic tools that can be used in addition to, or as an alternative to, radiographic methods has stimulated a growing interest in facial anthropometry. This is based on the mapping and measurement of the soft tissue of the face [1, 2]. To make better use of the potential of these methods, it is necessary to have reliable anthropometric data of reference populations, which is made possible through the analysis of a suitably large number of representative samples. It is therefore essential to have a highly standardized clinical-instrumental protocol that allows precise, repeatable, fast, and simple data acquisition that can be used for subjects of different ages and abilities to cooperate. This can then be used for the survey of suitably large samples that are representative of different subjects, and it should also be easy enough to carry out repeated mapping over time (i.e., monitoring of the spontaneous evolution of clinical cases or results obtained under given treatments).

**Figure 1:**
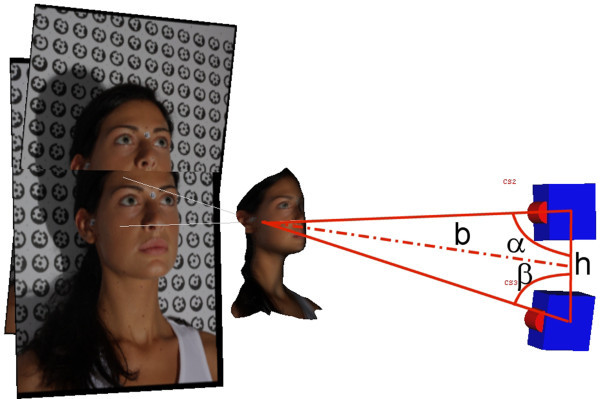
**The principle of triangulation as used for digital close-** **range photogrammetry.** CS2 and CS3, camera stations; *h, α, β,* distance and angles between the cameras; *b*, calculated *z* distance of the single point from the cameras.

There are various methods for the acquisition of data relating to the shape of a 3D object [3–6]. Digital close-range photogrammetry is suitable for use in medicine. The 3D information is obtained through the acquisition and comparison of a number of specific photographic images [7–9] that make use of the principle of triangulation, as shown schematically in Figure 1. For each couple of camera stations (CS2, CS3) rigidly positioned in the space, for each corresponding point *P*(*x*, *y*) in the two images, it is possible to calculate the third coordinate *z* as the value of *b* in the triangle, knowing the distance and angles between the cameras (*h*, *α, β*).

The photographs must be taken from at least two different positions, such that through the intersection of the virtual lines drawn from each of the cameras to the object (here, the face), it is possible to define the 3D coordinates of the points of interest and, therefore, the shape and size of the object [10]. The positions and angles of orientation of each of the cameras must be known for all of the pictures in a dataset [11].

**Figure 2:**
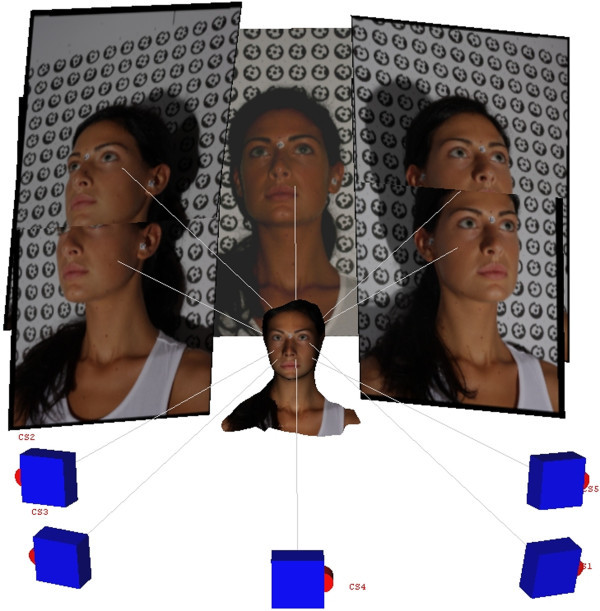
**The images captured with the use of five cameras.**

A 3D model of the facial soft tissue can then be obtained in the form of a point cloud, which provides a list of points described according to their spatial coordinates. Through the connection and recognition of the characteristics that bind these 3D points in the cloud, a mesh is created, i.e., a reconstruction of the face that consists of tiny polygons, usually triangles. A computer equipped with the photogrammetric software can then be used to process the images (Figure 2).

A new method of 3D scanning can be used to create dense 3D point clouds and to reconstruct a detailed virtual model of the surfaces, which is known as photo-based scanning. This process uses digital cameras in combination with algorithms that analyze digital images [11]. The algorithm for the processing of the scans compares image pairs on the basis of small portions (or patches), area by area, evaluating which patches correspond to one another. Once the optimal correspondence is defined, the position and orientation already computed for photographs are used to calculate the position of this patch in the 3D space. The accuracy and reliability of 3D measurements of facial soft tissue were evaluated by Swennen et al. [12] through comparisons of data obtained on the same sample of subjects using 3D computer tomography and 3D stereophotogrammetry. The measurement of the facial soft tissue with 3D computer tomography showed great accuracy, except for the landmarks of the hairline, the eyebrows, and the eyelids. In contrast, 3D stereophotogrammetry showed great accuracy and reliability, except for the bony landmarks. Thus, the combination of 3D computer tomography and 3D stereophotogrammetry for the analysis of facial soft tissue allows these problems related to the hairline, eyebrows, eyelids, and the bony landmarks to be overcome [12]. The study of Ghoddousi et al. [13] compared the accuracy of the mapping of facial soft tissue using 3D stereophotogrammetry, manual anthropometric measurements, and 2D photographs. These three methods showed good repeatability of measurements. The level of accuracy of the 3D stereophotogrammetric system was very satisfactory, both for the measurement of distances and for the mapping of surfaces. Indeed, the measurements obtained with the 3D systems were sufficiently accurate and reliable enough for clinical use [14].

In surface anthropometry, indirect methods (i.e., those without direct physical contact between the subject and the mapping system), such as photogrammetry, have several advantages over direct methods (i.e., those with direct physical contact needed). This can be seen when the sensors of the equipment used for direct methods can deform the facial surface during measurements, albeit only slightly, and thus contribute as a source of inaccuracy. Instead, photogrammetric methods are relatively insensitive to slight movements of the body, as the time of interaction with the subject is shorter, and therefore, this method is less influenced by any behavior of the subject. Moreover, some measurements, such as those relating to the eyes, are difficult to obtain with direct mapping methods, without causing discomfort or possible injury to the patient [12, 14, 15].

Previous studies investigated on the accuracy of the measurements made using photogrammetric face scanners. Rangel et al. [16] presented a study using 3dMD™ (Atlanta, GA, USA) stereophotogrammetric system: they obtained a 3D digital model having an average error of 0.35 mm with a standard deviation of 0.32 mm. Khambay et al. [17] validated a high-resolution 3D imaging system (Di3D™, Dimensional Imaging Ltd., Glasgow, Scotland, UK) using only ten landmarks on facial plaster casts, comparing the results with those obtained by a coordinate-measuring machine (CMM), reporting a system error of 0.2 mm and a reproducibility error of less than 0.5 mm. Winder et al. [18] used 18 landmarks on a mannequin head to validate a Di3D™ system, using comparison laser scans and digital caliper measures, having mean differences between measures of 0.62 mm. In 2010, Lübbers et al. [19] examined the precision and accuracy of the 3dMD™ system using 41 landmarks on a mannequin head: they report a mean global error of 0.2 mm (range 0.1 to 0.5mm). More recently, they [20] repeated the experiments using 61 landmarks on two real faces, reporting a mean global error of 0.41 mm (range 0 to 3.3 mm). Deli et al. [21, 22] compared the results achieved with various photogrammetric systems having different numbers and types of cameras. Galantucci et al. [23] recently also developed a simple photogrammetric system for automatic capture and measurement of facial soft tissues during movement: digital close-range photogrammetry was used to acquire the spatial coordinates of facial landmark points and track their movements.

In [24], the Face Shape Maxi5 3D photogrammetric scanner developed by Polishape 3D srl (Bari, Italy), the one used in this study, was validated in terms of reproducibility and accuracy. Measurements were taken over a set of 23 anthropological soft tissue facial landmarks marked on two different dummies, assessing photogrammetric software precision and system measurement accuracy. The operator error was measured by repeatedly digitizing landmarks on the 3D model, and it was around 0.059 mm. The reproducibility error was calculated by digitizing landmarks on two different occasions. The average Euclidean distance between the two matched sets of coordinates was thus computed, giving a result of approximately 0.090 mm. Each dummy was digitized for comparison using a CMM of documented accuracy (0.5 μm). The as-obtained landmark coordinates were considered as the ‘gold standard’. The system error mean value was then found to be equal to 0.425 mm, with a standard deviation of 0.142 mm. Therefore, accuracy results suggested that the 3D scanning system used in this work was reliable enough to capture the facial morphology for clinical and anthropological usage.

This methodology thus provides higher accuracy with respect to laser scanning (Vivid 910i, Minolta, Osaka, Japan; a method based on triangulation and widely used in the literature for 3D facial scans), which has an accuracy of ±0.38 and ±0.31 mm in the *X* and *Y* directions, respectively, and ±0.2 mm in the *Z* direction.

The aim of the study was to define a standardized clinical-instrumental procedure to perform such facial scanning and mapping with the use of a specific 3D photogrammetric methodology that allows the reconstruction of 3D digital models of the face. Thus, with the aid of dedicated computer software, this allows quantitative and qualitative anthropometric evaluations of the surface characteristics of the facial soft tissue.

## Methods

**Figure 3:**
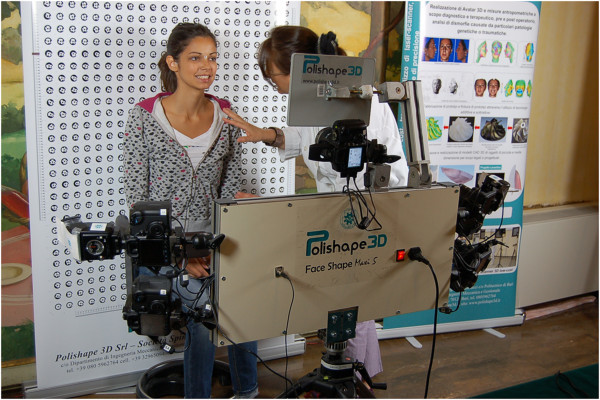
**Face Shape Maxi 5 of Polishape 3D (a spin-off company of Politecnico di Bari).**

The 3D photogrammetric method that was designed and implemented in the Laboratory of Rapid Prototyping and Reverse Engineering in Politecnico di Bari (Italy) using the equipment shown in Figure 3. The choice of photogrammetry over other optical scanning methods, such as laser or structured light, was done because photogrammetry is the only method that allows simultaneous acquisition of a large visual and angular field in a much shorter time (1/100 to 1/5,000 s) than with other techniques [3].

The equipment used included five high-definition digital single-lens reflex cameras with flash (Canon 40D, 10 Mpx) that were suitably mounted on a specific rigid structure and connected via computer for their simultaneous operation. The processing of the point clouds and the creation of the virtual 3D models were carried out through the use of photogrammetric reconstruction algorithms [25, 26]. The operational protocol designed for this facial scanning consisted of four phases defined as follows [27]:

**Figure 4:**
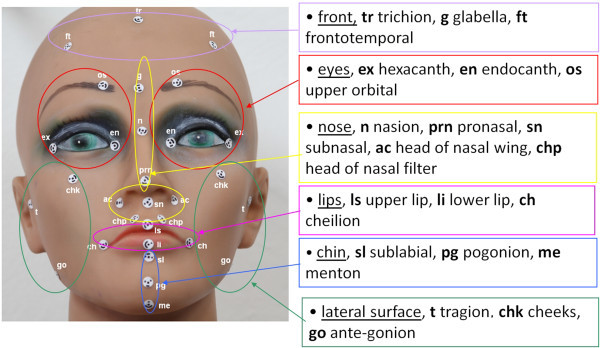
**Selection of the anthropometric landmarks** [28–32]**.**

**Figure 5:**
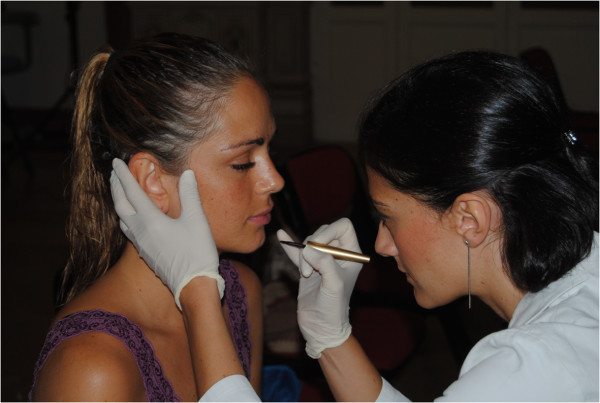
**Direct detection and marking of the anatomical landmarks.**

**Figure 6:**
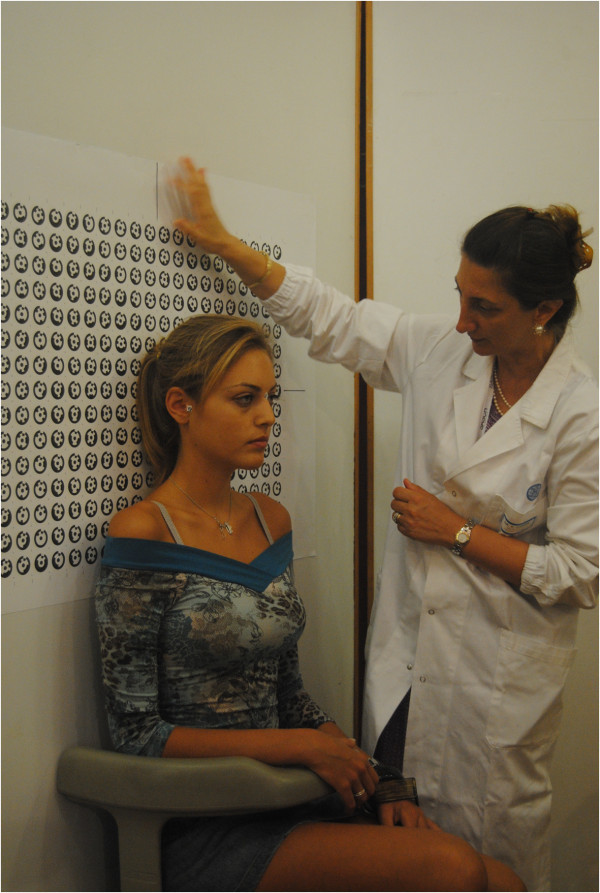
**Spatial positioning of the subject to be scanned.**

**Figure 7:**
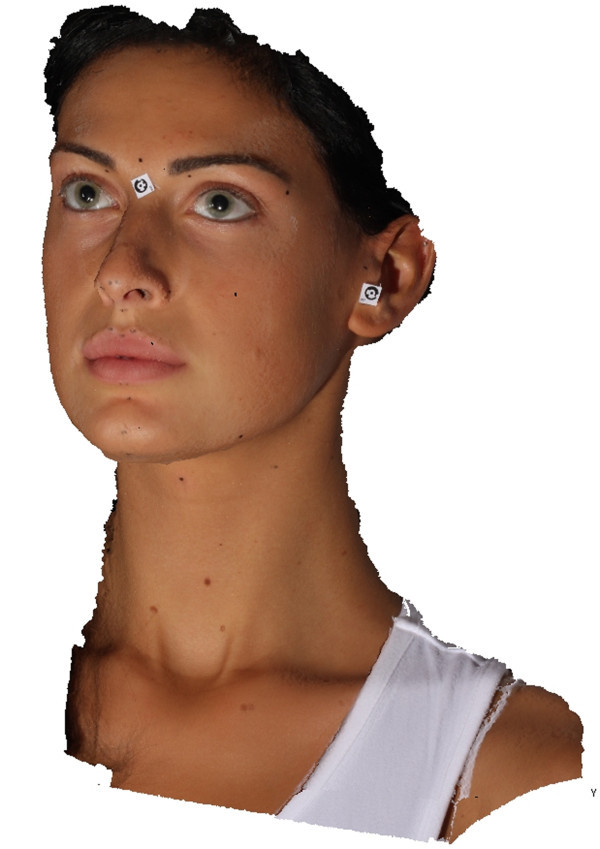
**Three-dimensional model of subject Miss TF.**

- *Phase 1*: *initial setup and preparation of the scanning device*. The cameras are mounted on an aluminum frame structure on a robust tripod. The structure contains synchronization and data communication electronics, electric tension transformers, and all of the connecting cables. This frame is designed and built to allow the correct orientation with respect to the face to be scanned (Figure 3). The structure is positioned at a distance of about 1 m from the subject and connected to a laptop computer used for the remote control of the cameras and to record the images acquired. Two multi-phosphorus fluorescent daylight lamps are positioned for the diffuse illumination of the subject and to facilitate the focusing operations. The background is provided with coded targets, in front of which the subject is seated on a stool that is height adjustable.
- *Phase 2*: *preparation of the face to be mapped*. The face must be free of make-up, with the hair back, to clearly and completely expose the parts of the face to be mapped and the natural texture of the skin. An operator identifies the anatomical skin landmarks on the face through direct method of inspection and palpation. In the present study, 33 landmarks were used, as selected from those according to Sforza et al. and Swennen and Farkas [28–32], and as illustrated in Figure 4. These were then marked by the same operator using hypoallergenic eyeliner to more easily locate the positions of these various anatomical landmarks (Figure 5). The operator then applies three especially made hypoallergenic coded targets to the face at points N’, T_dx, and T_sx. The main skin landmarks used are [29, 33–35] midpoints (trichion (Tr), glabella (G), skin nasion (N’), pronasal (Prn), subnasal (Sn), labialis superior (Ls), labial inferior (Li), sub labialis (Sl), skin pogonion (Pg’), skin menton (Me’)) and bilateral points (upper orbital (Os), frontotemporal (Ft), endocanthion (En), exocanthion (Ex), chresta philtri (Chp), cheilion (Ch), tragion (T’), nasal wing crest (Ac), skin antegonial point (Go’)).
- *Phase 3*: *spatial positioning of the subject to be scanned* (Figure 6). For the measurement of the scale during the scan, a specific reference sample is positioned on the subject. The subject is seated in front of the background of the set, which contains the coded targets, on the adjustable height stool positioned facing, and at about 1 m from, the photographic equipment. The head of the subject must be suitably framed by all of the cameras, both at the front and at the sides. It is advisable to use a mirror positioned directly above the cameras, which should be an integral part of the equipment, so that the subject can more easily comply with the conditions for the natural head position, which incorporates a natural expression of the soft tissue, with the eyes gazing into the distance. Three scans are carried out under different shooting conditions: the subject alone at rest (without any specific reference sample that would be useful for subsequent measurements) and two more scans with the inclusion of the reference sample that is applied directly on the subject: the first of these under conditions of rest, and the second, while smiling. The necessary instructions are provided to ensure that during the scanning, while resting, the subject retains their usual dental occlusion (so as not to alter their anthropometric measurements), with the lips at rest and maintaining a very natural facial expression; even the acquisition while the subject is smiling has to maintain the same conditions.
- *Phase 4*: *data processing*. Using the appropriate procedures, a very dense 3D cloud of the spatial points is obtained (potentially with millions of points). The 3D individual digital models are created from this 3D cloud (Figure 7). Finally, an automatic procedure for the extraction of the 3D models and the storage of the coordinates of the selected landmarks is also created.

This new clinical-instrumental protocol for photogrammetric acquisition was applied for the first time to acquire the faces of a sample of 66 female subjects (64 Caucasian, 1 Ethiopian, and 1 Brazilian), who are finalists in an Italian national beauty contest in 2010. Each subject explicitly expressed their free and voluntary participation in this study and allowed the 3D photogrammetric scanning of their face and related facial analyses. The 3D scanning of their faces was all carried out on the same day, having at least three different scans for each subject.

The software used for photogrammetric processing was PhotoModeler™ Scanner version 6 (Eos Systems Inc., Vancouver, BC, Canada), while for the development of the 3D models, Geomagic™ version10 (Morrisville, NC, USA) was used. PhotoModeler provides estimates of how precise the positions of the points computed are, using the processing algorithm.

On the basis of these coordinates, it is possible to identify a series of anthropometric measurements and angles that can provide a guide and tool for surgeons and orthodontists involved in the reevaluation of female faces. The *i*th linear distance was calculated as 1

where *x*, *y*, and *z* are the spatial coordinates of the *j* and *k* landmarks.

The (rp-rq)_*ith*_ angle measurement was calculated (in sexagesimal degrees) using Equation 2:2

wherewhere *x*, *y*, and *z* are the coordinates of two points on the *r* line.

- mp and np are the slopes of the rp line, and mq and nq are the slopes of the rq line.
- rp and rq are the lines that form the angle under query.

## Results and discussion

### Results

This new protocol is very fast for the acquisition of the face. There is the need for about 1 h for the first phase of the protocol (the preparation of the scanning system and of the set must be performed only once for the installation of the equipment, and then it can work for the acquisition of the whole sample to be scanned in that environment). The subject preparation (second phase) takes about 1.5 min per subject; the correct subject positioning and the acquisition of three scans (third phase) also takes for about 1.5 min per subject. The time taken by the system for each acquisition is actually 6 ms, as the duration of the flash. The fourth phase of data processing requires about 15 min by a skilled operator (often an engineer) for the manipulation of the 3D images. From previous studies using this methodology [21, 24–26], the average accuracy of the measurements made onto the digital model, referring to the entire surface of the face, was 0.5 to 0.6 mm, while for the individual points (the coded targets), the measurement accuracy obtained was 0.03 mm in the *X* and *Y* directions and up to 0.15 mm in the *Z* direction.

These three scans were then used to build the 3D digital models under the different shooting conditions of the scanning subject (subject under resting conditions without and with references samples and while smiling).

These 3D digital models allow the automatic extraction of the precise spatial coordinates of the anatomical landmarks selected (Figure 7). As indicated above, the direct pre-marking of the selected landmarks on the face makes these easily identifiable and uniquely recognizable on the virtual model of the face, allowing extremely precise anthropometric measurements [22, 24, 26].

The standardization of the methodology made it possible to define the samples evenly, recreating the same conditions of data acquisition for all the subjects. Following these requirements for each of the four phases of the protocol provided good repeatability for data acquisition [3, 24, 36], which was associated with excellent mapping precision, which is an intrinsic characteristic of this methodology [24, 37].

**Figure 8:**
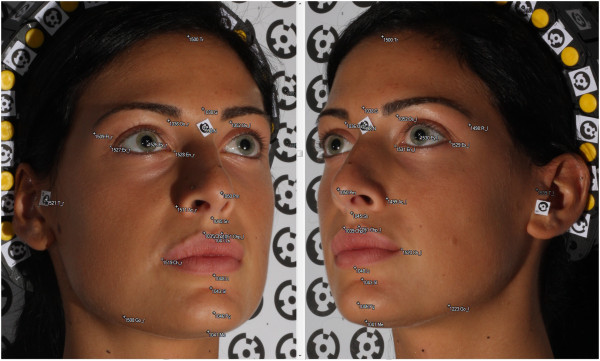
**Anthropometric landmarks of subject Miss TF.**

Figure 7 shows the development of the 3D model of one of the subjects of the sample analyzed (Miss TF) and specifically the subject who should be the most attractive of all as she won the final of the 2010 competition. Figure 8 shows the 3D spatial mapping of the anthropometric landmarks obtained via computer on the digital images of subject Miss TF.

The 3D coordinates of the anatomical landmarks and the estimation of the accuracy of each measurement for subject Miss TF are listed in Table 1.The linear and angular measurements and the measurement ratios of subject Miss TF are given in Tables 2 and 3.

**Table 1:** **Three**-**dimensional coordinates of the anatomical landmarks and estimation of the precision of each measure**

Landmark	***X***(mm)	***Y***(mm)	***Z***(mm)	Precision vector length (mm)
Ac_l	14.20	−41.65	−1.28	1.11
Ac_r	−15.87	−42.59	−3.40	1.02
C_c	4.00	−123.54	−57.77	0.17
C_l	38.02	−116.39	−74.18	0.18
C_r	−24.05	−116.60	−77.28	0.18
Ch_l	21.43	−70.05	−2.50	1.08
Ch_r	−23.98	−70.81	−5.30	1.00
Cph_l	3.57	−57.67	10.74	0.17
Cph_r	−6.58	−57.83	10.11	0.17
En_l	14.77	−10.92	−12.42	0.20
En_r	−13.38	−10.98	−14.11	1.06
Ex_l	43.04	−9.20	−17.00	0.17
Ex_r	−40.14	−8.47	−23.84	1.06
Ey_l	30.24	−6.14	−9.69	0.12
Ey_r	−27.06	−6.68	−13.07	0.12
Ft_l	53.32	−0.61	−25.22	1.14
Ft_r	−50.12	−0.89	−32.44	1.07
G	−0.66	10.21	−0.22	0.17
Go_l	38.57	−100.48	−26.91	1.09
Go_r	−34.99	−99.86	−30.97	1.02
Li	−0.55	−80.01	8.82	0.16
Ls	−1.06	−60.00	10.98	0.16
Me	0.41	−109.10	−1.48	0.16
N	0.00	0.00	0.00	0.05
Os_l	18.58	4.82	−5.22	0.17
Os_r	−17.23	4.33	−8.46	0.17
Pg	0.16	−99.49	6.63	0.16
Prn	−2.34	−38.18	23.19	0.16
Sl	0.53	−85.94	2.90	0.16
Sn	−1.77	−49.80	10.32	0.17
T_l	72.84	−42.17	−76.74	0.13
T_r	−57.88	−31.26	−83.51	0.12
Tr	0.96	53.57	−18.75	0.18
*Mean value*				*0.43*

Data for Miss TF.

**Table 2:** **Linear and angular measurements and the measurement ratios of subject Miss TF**

Landmarks involved	Name	Type and units	Value
N-Pg	Facial line	Distance (mm)	99.7
N-P	Nasion-midpoint of facial line	Distance (mm)	49.9
P-Pg	Midpoint of facial line-nasion	Distance (mm)	49.9
P-M(T_r-T_l)	Midpoint of facial line-midpoint of tragi	Distance (mm)	84.8
N-Ls	Nasion-Ls upper lip	Distance (mm)	61.0
Ls-Prn	Ls upper lip-pronasal	Distance (mm)	25.0
N-M(T_r-T_l)	Nasion-midpoint of tragi	Distance (mm)	88.5
Prn-M(T_r-T_l)	Pronasal-midpoint of tragi	Distance (mm)	103.8
Ls-M(T_r-T_l)	Upper lip-midpoint of tragi	Distance (mm)	94.4
Pg-M(T_r-T_l)	Pogonion-midpoint of tragi	Distance (mm)	107.3
Tr-Sn	Trichion-subnasal	Distance (mm)	107.4
Tr-N	1° Third facial height	Distance (mm)	56.8
N-Sn	Anterior upper facial 2° third height	Distance (mm)	50.9
Sn-Me	Anterior upper facial 3° third height	Distance (mm)	60.5
Ex_r-En_r	Eye r	Distance (mm)	28.6
Ex_l-En_l	Eye l	Distance (mm)	28.7
En_r-En_l	Eye distance	Distance (mm)	28.2
Ey_r-Ey_l	Eye pupillar distance	Distance (mm)	57.4
Ch_r-Ch_l	Oral length	Distance (mm)	45.5
Ac_r-Ac_l	Nasal width	Distance (mm)	30.2
1.5*(Ac_r-Ac_l)	1.5 × Nasal width	Distance (mm)	45.2
Ex_r-N	Exocantion r-nasion	Distance (mm)	47.4
N-Ex_l	Nasion-exocantion l	Distance (mm)	47.2
Ex_r-Ex_l	Upper facial width	Distance (mm)	83.5
Go_l-Go_r	Lower facial width	Distance (mm)	73.7
Pg-M(Go_l-Go_r)	Mandibular corpus length	Distance (mm)	35.6
N-Prn	Nasion-pronasal	Distance (mm)	44.7
Prn-Pg	Pronasal-pogonion	Distance (mm)	63.6
N-Sn	Anterior upper facial	Distance (mm)	50.9
Sn-Pg	Anterior lower facial height	Distance (mm)	49.9
T_r-T_l	Middle facial width	Distance (mm)	131.4
Sn-(T_r-T_l)	Middle facial depth	Distance (mm)	91.9
Ch_l-Ch_r	Mouth width	Distance (mm)	45.5
Ls-(Prn-Pg)	Upper lip to E-line distance	Distance (mm)	6.1
Li-(Prn-Pg)	Lower lip to E-line distance	Distance (mm)	3.0
Sl-N	Sublabial-nasion	Distance (mm)	86.0
Prn-Sn	Pronasal-subnasal	Distance (mm)	17.3
Sn-Ls	Subnasal-upper lip	Distance (mm)	10.2
Ls-Pg	Upper lip-pogonion	Distance (mm)	39.7
T_r-Prn	Tragion r-pronasal	Distance (mm)	120.5
T_l-Prn	Tragion l-pronasal	Distance (mm)	125.1
T_r-Pg	Tragion r-pogonion	Distance (mm)	127.1
T_l-Pg	Tragion l-pogonion	Distance (mm)	124.6
T_r-N	Tragion r-nasion	Distance (mm)	106.3
T_l-N	Tragion l-nasion	Distance (mm)	113.9
Go_l-Pg	Gonion l-pogonion	Distance (mm)	51.0
Pg-Go_r	Pogonion-gonion r	Distance (mm)	51.5
T_r-Go_r	Tragion r-gonion r	Distance (mm)	89.4
T_l-Go_l	Tragion l-gonion l	Distance (mm)	84.0
T_l-Sn	Trago_left-subnasal	Distance (mm)	114.9
Sn-T_r	Subnasale-trago_right	Distance (mm)	107.4
Ls-Li	Vermillion height	Distance (mm)	20.1
Sl-Pg	Sublabial-pogonion	Distance (mm)	14.1

**Table 3:** **Angular measurements and the measurement ratios of subject Miss TF**

Landmarks involved	Name	Type and units	Value
N-Sn-Pg	Facial convexity excluding the nose	Angle (deg)	163.5
Sl-N-Sn	Maxillary prominence	Angle (deg)	10.0
Prn-Sn-Ls	Naso-labial	Angle (deg)	128.4
(Sn-Ls)^(Sl-Pg)	Interlabial	Angle (deg)	167.1
N-Prn-Pg	Nasion-pronasal-pogonion	Angle (deg)	133.3
T_l-Prn-T_r	Tragion l-pronasal-tragion r	Angle (deg)	64.6
T_l-Pg-T_r	Tragion l-pogonion-tragion r	Angle (deg)	62.9
T_l-N-T_r	Tragion l-nasion-tragion r	Angle (deg)	73.1
Sn-N-Prn	Subnasale-nasion-pronasal	Angle (deg)	19.6
T_l-Go_l-Pg	Tragion l-gonion l-pogonion	Angle (deg)	133.1
T_r-Go_r-Pg	Tragion r-gonion r-pogonion	Angle (deg)	126.8
Go_l-Pg-Go_r	Lower face convexity	Angle (deg)	91.9
T_l-Sn-T_r	Middle face convexity	Angle (deg)	73.4
Ex_r-N-Ex_l	Upper facial convexity	Angle (deg)	56.2
F (Pg-P-M(T_r-T_l))	Facial angle	Angle (deg)	102.6
Mf (Pg-N-Ls)	Maxillo-facial angle	Angle (deg)	6.6
Nm (Ls_P-P_M(T_r-T_l))	Naso-maxillary angle	Angle (deg)	110.5
Na (N-M(T_r-T_l)-Prn)	Nasal angle	Angle (deg)	25.3
Mx (Prn-M(T_r-T_l)-Ls)	Maxillary angle	Angle (deg)	13.5
Mn (Pg-M(T_r-T_l)-Ls)	Mandibular angle	Angle (deg)	21.5
Tv (N-M(T_r-T_l)-Pg)	Total vertical angle	Angle (deg)	60.3
(T_r-T_l)/(N-Pg)	Middle facial width to facial height	Ratio	1.32
(N-Sn)/(N-Pg)	Nasion-subnasal/nasion-pogonion	Ratio	0.51
(Sn-Pg)/(N-Pg)	Subnasale-pogonion/nasion-Pogonion	Ratio	0.50
(Tr-N)/(Tr-Sn)	Trichion-nasion/trichion-subnasal	Ratio	0.53
(Sn-Pg/N-Sn)x100	Lower to upper facial height	Percentage	98.0%

### Discussion

The protocol that we have developed here has highlighted some key elements that have to be met to ensure the full and correct performance and reliability of the measures.

It is essential that the system has full synchronization of the shutters of the cameras. In *phase 1* it is important to assure the correct positioning of the system and a good illumination of the face. In *phase 2*, for a good repeatability and comparability of measurements, the positioning of the anatomical landmarks must be done very accurately. For multiple scanning, the operations of this second phase must be performed by the same operator to provide a uniform standard of the mapping (the same intra-operator error in all of the mapping carried out) and also to avoid incorporating interoperator error into the methodology of the mapping (this error must be considered in the case of measurements taken by different operators). During *phase 3*, close attention must be paid to the correct positioning of the head. Indeed, although a 3D model is theoretically insensitive to the position of the head, this is not actually true because the distribution of the soft tissue of the face can change under the directional force of gravity. During this third phase, close attention must also be paid to the correct facial expression as the distribution of the soft tissue of the face can change as a result of the facial expression itself. In *phase 4*, it is crucial to make use of suitable hardware and software for the processing of the point clouds as these contain huge amounts of data. The automatic mapping of the spatial coordinates of the soft tissue pre-marked landmarks of the 3D virtual model allows for numerous measurements to be made for subsequent post-processing (e.g., lengths, angles, linear and angular ratios). These measurements are useful to obtain multiple high-precision biometric measurements.

The estimations of the accuracy of measurements are in line with what is generally considered to be acceptable for facial anthropometric measurements (1.5 mm) and with what has been obtained in previous studies (0.5 to 0.6 mm) [21, 22, 24, 26]. For the subject illustrated as an example, the average of the estimations of precision was 0.43 mm. The analysis of the data shows that the greatest uncertainty in the measurement (i.e., the greatest value of the precision vector) was associated with some points that were not marked directly on the face with the eyeliner but were identified on the virtual model after the 3D reconstruction (Ex, En, Ch) and with some points that were detected by only two of the cameras (Ac, Ft, Go). The smallest uncertainty was associated with the estimate of the position of the N-nasion (0.05 mm), while the largest uncertainty was associated with Ft_l, the left frontotemporal skin landmark (1.14 mm).

It is interesting to evaluate whether the measures correspond to average values or to measures of attractiveness. As an example, we have reported here the analysis of subject Miss TF, in terms of the neoclassical canons of facial proportion [38] and the evaluation of the profile according to Peck and Peck [39]. The neoclassical canons that were used for the comparisons were as follows [38]:

- *Vertical proportions*. The three facial sections of Tr-N, N-Sn, and Sn-Me must have the same length.
- *Naso-aural proportions*. The distances N-Sn and Sa-Sba must be the same.
- *Naso-aural slope*. The slope of the nose should be equal to the slope of the ear.
- *Horizontal proportions*. The interocular width of En_r-En_l must be equal to the width of the eye as En_r-Ex_r and En_l-Ex_l.
- *Orbito-nasal proportions*. The interocular width of En_r-En_l must be equal to the width of the nose as Ac_r-Ac_l.
- *Nose-mouth proportions*. The width of the nose of Ac_r-Ac_l must be two thirds of the width of the mouth as Ch_r-Ch_l.
- *Nose-face proportions*. The width of the nose of Ac_r-Ac_l must be one quarter of the width of the face as Zy_r-Zy_l.

**Figure 9:**
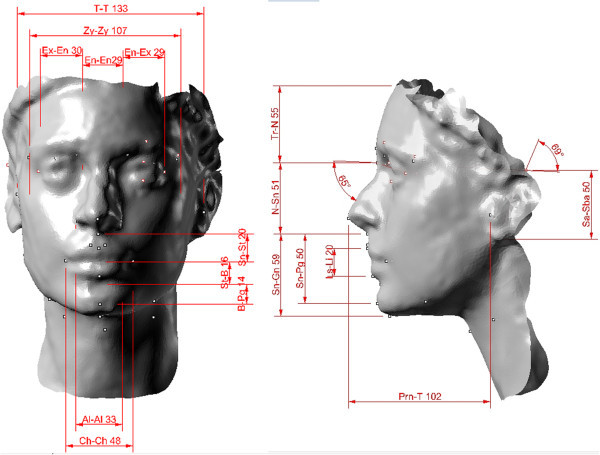
**Measurements of the neoclassical canons of Miss TF.**

From the analysis of the data obtained from the measurements taken on Miss TF (Figure 9), it is clear that the naso-aural, horizontal, and nose-mouth proportions of her face are very close to the neoclassical canons (Table 4). According to Farkas et al. [38], the orbito-nasal proportions of the lower third of her face, the naso-aural slope, and the proportions of the lower third of her face deviate slightly from the neoclassical canons. Then, according to Piero della Francesca and Durer reported in Farkas [38], the vertical and nose-facial proportions and those of the lower third of her face deviate substantially from the neoclassical canons.

**Table 4:** **Evaluation of the measurements of the neoclassical canons of Miss TF**

Neoclassical canons[30]	Measure	Description	Value for Miss TF
Vertical proportion: the three facial sections must have the same length	Tr-N	1° Third facial height	55 mm
	N-Sn	Anterior upper facial 2° third height	51 mm
	Sn-Me	Anterior upper facial 3° third height	59 mm
Naso-aural proportion: the distances N-Sn and Sa-Sba must have the same value	N-Sn	Anterior upper facial 2° third height	51 mm
	Sa-Sba		50 mm
Horizontal proportion: the eye distance must be equal to the ocular width	Ex_r-En_r	Eye r	30 mm
	Ex_l-En_l	Eye l	29 mm
	En_r-En_l	Eye distance	29 mm
Orbito-nasal proportion: the eye distance width must be equal to the nasal width	En_r-En_l	Eye distance	29 mm
	Ac_r-Ac_l	Nasal width	33 mm
Nose-mouth proportion: nasal width should be two-thirds of the oral length	Ch_r-Ch_l	Oral length	48 mm
	1.5 × (Ac_r-Ac_l)	1.5 × Nasal width	49.5 mm
Nose-face proportion: the nasal width must be a quarter of the facial width	0.25 × (Zy_r-Zy_l)	0.25 × Face width	27 mm
	Ac_r-Ac_l	Nasal width	33 mm
Proportions of the lower third of the face (Pierodella Francesca) [38]	Sn-Sto	19.7=1/3 of Sn-Me (59 mm)	20 mm
	Sto-Sl	19.7=1/3 of Sn-Me (59 mm)	16 mm
	Sl-Me	19.7=1/3 of Sn-Me (59 mm)	23 mm
Proportions of the lower third of the face [38]	Sn-Sto	18.4 =31.2% of Sn-Me (59 mm)	20 mm
	Sto-Sl	14.9 =25.2% of Sn-Me (59 mm)	16 mm
	Sl-Me	25.7 =43.6% of Sn-Me (59 mm)	23 mm
Proportions of the lower third of the face (Durer) [38]	Sn-Sto	14.75 =25% of Sn-Me (59 mm)	20 mm
	Sto-Sl	14.75 =25% of Sn-Me (59 mm)	16 mm
	Sl-Pg	14.75 =25% of Sn-Me (59 mm)	14 mm
	Pg-Me	14.75 =25% of Sn-Me (59 mm)	9 mm
Naso-aural inclination: the angle of the nose should be equal to the inclination of the ear	Angle of the nose	Nose inclination	65°
	Angle of the ear	Inclination of the ear	69°

The analysis of the measurements of Miss TF performed according to a profile analysis of Peck and Peck [39] (Table 5) shows that her facial angle and jaw angle are very close to the average values reported by their analysis of attractive women. Her nasal angle and the maxillo-facial angle differ slightly, while her mandibular, nasal-maxillary, and total vertical angle show the largest deviations from the average values of Peck and Peck [39].

**Table 5:** **Evaluation of measures in the analysis of the profile of Miss TF**

Measure	Description	Peck and Peck mean value (deg)	Miss TF (deg)
F (Pg-P-M(T_r-T_l))	Facial angle	102.5	102.6
Mf (Pg-N-Ls)	Maxillo-facial angle	5.9	6.6
Nm (Ls_P-P_M(T_r-T_l))	Naso-maxillary angle	106.1	110.5
Na (N-M(T_r-T_l)-Prn)	Nasal angle	23.3	25.3
Mx (Prn-M(T_r-T_l)-Ls)	Maxillary angle	14.1	13.5
Mn (Pg-M(T_r-T_l)-Ls)	Mandibular angle	17.1	21.5
Tv (N-M(T_r-T_l)-Pg)	Total vertical angle	54.5	60.3

According to Peck and Peck [39].

## Conclusions

The protocol described here allows the study of anthropometric characteristics of faces, making it easy to apply various methods of facial morphometric analysis on 3D digital models, both in profile and as a frontal view. The analysis of the neoclassical canons [38, 40] and the analysis of profiles according to Peck and Peck [39] are also described here, and it would also be easy to perform further esthetic analyses, such as of the profile according to Bütow [41], the cephalometric analysis of soft tissue (ACTM) according to Arnett and McLaughlin [33], the 3D facial morphometry method according to Ferrario et al. [42], and the method of Baik et al. [43] to mention a few.

The direct pre-marking of the anatomical landmarks on the face makes it easy to automatically recognize their 3D positions on the 3D digital model as these are clearly visible and easily identifiable with respect to the other structures of the face, and this improves the goodness of the measurement of the coordinates of these points. The speed of this methodology made it possible to apply it in a facial mapping situation that was very particular, i.e., the national final of a popular Italian beauty contest. This provided data on the anthropometric characteristics of the faces of contestants who were considered to be the more attractive, as they had passed the initial wide selection made by juries across the whole of Italy. The selection had thus started with a sample size of about 20,000 girls to arrive at this sample of only 66 finalists in 2010. Indeed, it is the speed of the method that makes it relatively easy to apply in different facial mapping situations, on large sample groups or for extensive clinical trials. This can also be applied to different age groups and in the case of subjects who cannot cooperate fully due to problems relating to age or the presence of various handicaps. For the subjects being examined, this methodology appears to be well accepted as it is noninvasive, fast (it takes very little time for the subject), and easy to perform (the collaboration requested from the patient is minimal).

### Consent

Written informed consent was obtained from the patient for the publication of this report and any accompanying images.
